# Use of the HOPE score to assess survival outcome of hypothermic cardiac arrest selected by ECLS rewarming

**DOI:** 10.1186/s13049-025-01445-9

**Published:** 2025-07-28

**Authors:** Nicolas Hall, Jessika Métrailler-Mermoud, Valentin Rousson, Chloé Conforti, Amélie Dupasquier, Pierre-Nicolas Carron, Silke Grabherr, Bettina Schrag, Matthias Kirsch, Cheyenne Falat, Dominique Delay, Vincent Frochaux, Mathieu Pasquier

**Affiliations:** 1 Department of Emergency Medicine, Lausanne University Hospital and University of Lausanne, BH09, 1011 Lausanne, Switzerland; 2 Emergency Service, Valais Hospital, 1951 Sion, Switzerland; 3 Center for Primary Care and Public Health (Unisanté), Lausanne, Switzerland; 4 Lausanne University School of Medicine, Lausanne, Switzerland; 5 University Center of Legal Medicine, Lausanne - Geneva, Lausanne University Hospital and University of Lausanne, Geneva University Hospital and University of Geneva, Geneva, Switzerland; 6 Legal Medicine Service, Hospitals Central Institute (ICH), Sion, Switzerland; 7 Department of Cardiac Surgery, Lausanne University Hospital, Lausanne, Switzerland; 8 Department of Emergency Medicine, University of Maryland School of Medicine, Baltimore, MD USA; 9 Department of Cardiac Surgery, Valais Hospital, 1951 Sion, Switzerland

**Keywords:** Accidental hypothermia, Cardiac arrest, ECLS, Environmental hypothermia, HOPE score, Benchmarking

## Abstract

**Background:**

We studied adult hypothermic cardiac arrest (CA) patients admitted to a University Hospital (UH) and a Regional Hospital (RH) for whom Extracorporeal Life Support (ECLS) was implemented. We used the HOPE score to estimate individual survival probabilities and to compare overall results between hospitals.

**Methods:**

We included hypothermic CA patients who underwent ECLS between 2000 and 2022. We assessed the predicted survival probabilities by calculating the HOPE scores, both at individual and hospital levels. We assessed the performance of a HOPE score cutoff of 10% in predicting survival to hospital discharge, as ECLS rewarming is currently recommended when the HOPE is ≥ 10%. We also assessed the utility of the HOPE score in evaluating and comparing patient management within and between two hospitals.

**Results:**

In the 46 patients with successful ECLS implementation, a HOPE score < 10% would have contraindicated and therefore prevented futile ECLS rewarming procedures for 17 patients (37%) who did not survive, while finding that ECLS was indicated for 100% of survivors. The observed survival rate was 24% (UH: 35%, RH: 11%) whereas the HOPE score predicted a survival rate of 35% (UH: 41%, RH: 26%), suggesting underperformance of ECLS rewarming among both hospitals. The difference of survival between the two hospitals was not statistically significant.

**Conclusions:**

This study confirmed the utility of the HOPE score in estimating individual survival probabilities. The HOPE score may also be used to estimate the overall survival rate in a patient cohort, enabling internal quality-control and outcome results comparisons between different settings.

**Supplementary Information:**

The online version contains supplementary material available at 10.1186/s13049-025-01445-9.

## Background

Accidental hypothermia is defined as an involuntary drop in core temperature below 35°C. Hypothermic cardiac arrest (CA) generally occurs at a core temperature below 30 °C in healthy patients, although it has also been described in elderly and patients with multiples comorbidities with core temperatures between 30 and 32 °C [1–4]. Extracorporeal life support (ECLS) by using extracorporeal membrane oxygenation (ECMO) is the current treatment of choice for hypothermic CA. However, hospitals have limitations in the number of hypothermic CA patients whose resuscitation can be supported by ECLS. Its indication should be carefully assessed using appropriate decision criteria, and its use should be well-considered, also due to economic considerations. The HOPE score estimates the probability of survival to hospital discharge after ECLS rewarming for patients with hypothermic CA, based on six variables available at hospital admission: age, sex, mechanism of hypothermia, cardiopulmonary resuscitation (CPR) duration, serum potassium and core temperature [5, 6]. The 2021 European Resuscitation Council (ERC) guidelines for cardiac arrest in special circumstances have integrated the HOPE score into the accidental hypothermia management algorithm. The HOPE score calculation should be factored into the decision of whether to rewarm [3, 5, 6].

Our goal was to study hypothermic CA patients admitted to two different hospitals for whom ECLS was indicated. We compared patient characteristics, treatment, and outcomes between hospitals. The HOPE score was calculated retrospectively to explore potential differences in survival outcome case-mix, and hospital results were evaluated by comparing patient outcomes to expected survival probabilities. Post-mortem observations, when available, were also analysed.

## Methods

### Study population

This retrospective observational study was conducted in two Swiss centres: the University Hospital of Lausanne (UH) and the Valais Hospital, a regional hospital (RH) in Sion. We included adult patients over the age of 18 who presented with hypothermic CA at hospital admission (core temperature below 32 °C) who were rewarmed using ECLS. We excluded patients with return of spontaneous circulation (ROSC) before ECLS initiation. The inclusion period was January 1, 2000, to June 29, 2022.

### Data collection

The study and data collection were approved by the Cantonal ethics committee for human research (CER-VD), Switzerland (N° 2022-01206). An anonymised data transfer agreement between the two participating hospitals was obtained.

Clinical data were extracted manually from the prehospital charts and the hospital medical reports. We collected the following demographic, biological, and situational parameters: age, sex, mechanism of hypothermia, presence of signs of trauma, presence of a witnessed CA, CPR duration (defined as the time from initiation of CPR until initiation of ECLS), on-site and in-hospital core temperature measurement, initial rhythm of CA, serum potassium, and blood gas values at hospital admission (arterial or venous, depending on the sampling site). The mechanism of hypothermia was categorized as asphyxia-related (avalanche victims with head fully covered by snow or submersion victims with head fully covered by water, in CA at the time of extrication) or non-asphyxia-related (cold exposure or water immersion without high suspicion for hypoxemia prior to hypothermia) [5, 7]. We collected the following data related to the management of hypothermic CA: decision criteria leading to the use of ECLS rewarming, modality of ECLS rewarming (venoarterial ECMO [VA-ECMO] or cardiopulmonary bypass [CPB]), occurrence of ROSC, VA-ECMO duration (defined as the time from ECLS initiation to ECLS termination), and length of stay in the intensive care unit (ICU). The patient outcome data included: survival at hospital discharge and the presence of neurological sequelae assessed by the Cerebral Performance Categories (CPC) scale. A CPC of 1 or 2 was considered a favourable neurological outcome [8–10].

The University Centre of Legal Medicine of Lausanne and the Forensic Medicine Institute of Sion provided us with forensic data, collected thanks to an agreement with the corresponding prosecutor’s office. These data were obtained in the form of extracts from the forensic report addressed to the prosecutor, based on the results of all post-mortems examinations: external examination and complete forensic autopsy, including histology findings, clinical chemistry, and post-mortem whole body computed tomography (PMCT).

### Primary outcome

The primary study outcome was the accuracy of the retrospectively calculated HOPE score to predict hospital discharge after ECLS rewarming, both at the individual level and hospital levels, to assess the survival outcome and to compare the results of the two hospitals in managing hypothermic cardiac arrest patients selected for ECLS rewarming. The expected HOPE score probabilities of survival per hospital were obtained by summing all individually calculated HOPE scores and dividing by the total number of patients who received ECLS rewarming.

### Secondary outcome

The secondary study outcome was the comparison between potassium cutoff (the historical decision criterion) and the HOPE score cutoff to decide whether ECLS rewarming was indicated. According to guidelines from 2001 to 2021, a serum potassium greater than 12 mmol/L contraindicated ECLS rewarming, with a lower cutoff of 8 mmol/L used from 2013 to 2022 to contraindicate ECLS among avalanche victims [1, 11–13]. A HOPE score threshold of <10% was considered to be a contraindication to ECLS rewarming. This cutoff has been suggested to minimize the number of futile ECLS rewarming attempts, while not missing patients who would benefit from ECLS rewarming [5, 6, 14, 15]. We also analysed the forensic reports of patients who did not survive despite ECLS rewarming attempt.

### Statistical analysis

The extracted data were entered on an Excel spreadsheet (Microsoft, Redmont, WA, USA) and exported to Stata v20 (Stata Corporation, College Station, TX, USA) for analysis. Continuous variables were reported as mean ± standard deviation (SD) and compared with Student’s t-tests when normally distributed, and as medians and interquartile ranges using Mann-Whitney U tests when not normally distributed. Categorical data were expressed as numbers and percentages and analysed using the chi-square test or Fisher’s exact test, as appropriate. Raw proportions of survivors between two hospitals were compared using a chi-square test. In order to get an adjusted comparison, to take into account that the mix of patients might be different in each hospital, we compared the number of observed survivors with the number of expected survivors, where the latter was obtained by summing the HOPE survival probabilities. For example, if two patients have a survival probability of 0.5, we expect one of them to survive. Thus, the difference between an expected and an observed number of survivors can be interpreted as a deficit of survivors, so that hospitals can be fairly compared in this regard. We denoted by ${o}_{ih}$ the observed survival status after ECLS rewarming for each patient $i$ in hospital $h$ (${o}_{ih}=1$ for survivors, ${o}_{ih}=0$ for non-survivors), and by ${e}_{ih}$ the expected survival probability calculated at hospital arrival according to HOPE. The terms ${po}_{1}$ and ${po}_{2}$ were the observed survival proportions in the two hospitals (i.e. the mean of the ${o}_{i1}$ and the mean of the ${o}_{i2}$, respectively). The terms ${pe}_{1}$ and ${pe}_{2}$ were the corresponding expected survival proportions (i.e. the mean of the ${e}_{i1}$ and the mean of the ${e}_{i2}$, respectively). The deficit of survivors in hospital $h$ was calculated as ${d}_{h}={pe}_{h}-{po}_{h}$. Similar to a logrank test, our test for the statistical significance of a deficit ${d}_{h}$ was based on test statistic ${n}_{h}{d}_{h}^{2}/mean({p}_{ih}(1-{p}_{ih}))$, where ${n}_{h}$ denotes the sample size of patients from hospital $h$, to be compared with a chi-square distribution with 1 degree of freedom. An adjusted comparison of proportions of survivors between the two hospitals was then obtained as ${d}_{1}-{d}_{2}=\left({pe}_{1}-{po}_{1}\right)-\left({pe}_{2}-{po}_{2}\right),$ corresponding to a mean comparison of ${e}_{ih}-{o}_{ih}$ in the two hospitals.

## Results

**Fig. 1:**
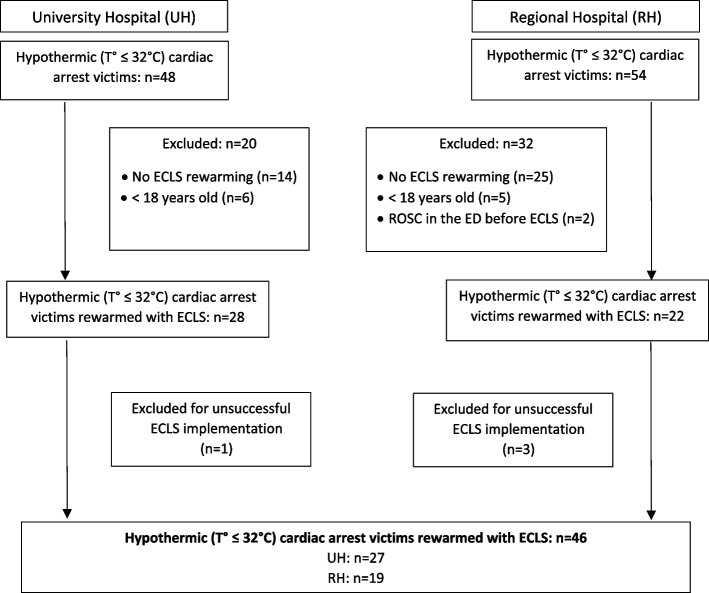
Flowchart of the study patients. Patients with hypothermic cardiac arrest at hospital admission (January 1, 2000, to June 29,2022). Abbreviations: CA, cardiac arrest; ECLS, extracorporeal life support; ED, emergency department; RH, Regional Hospital; ROSC, return of spontaneous circulation; UH, University Hospital

We identified 50 adult patients who presented in hypothermic cardiac arrest in whom ECLS rewarming was attempted. Among them, ECLS implementation was unsuccessful for 4/50 (8%) patients, 3/22 (14%) at the RH and 1/28 (4%) at the UH (*p*=0.193). This left 46 patients rewarmed with ECLS for analysis (Fig. 1).

Twenty-five victims (54%) were male. The mean age was 49 years (SD 16). The mechanisms of hypothermia were different between the two hospitals (*p*=0.015), with exposure being the predominant mechanism at the UH (*n*=17, 63%) and avalanche being the predominant mechanism at the RH (*n*=10, 53%). The initial mean core temperature measured in the prehospital setting was 23.7°C (SD 4.1) and measured at hospital admission was 24.0°C (SD 3.7). The prehospital data and characteristics at hospital admission of the study population are presented in Table 1 (with supplementary details in Additional file 1) and Table 2.

**Table 1:** Prehospital characteristics of patients who received ECLS rewarming (*n*=46)

	Missing	Overall (*n*=46)	University Hospital (*n*=27)	Regional Hospital (*n*=19)	*p*-value
Demographic					
Age^a^ (years), mean (SD)	0	49 (16)	48 (17)	50 (16)	0.66
Sex^a^ male, n (%)	0	25 (54)	14 (52)	11 (58)	0.69
Mechanism					
Asphyxia^a^, n (%)	0	20 (43)	9 (33)	11 (58)	0.10
Trauma, n (%)	4	22 (52)	11 (48)	11 (58)	0.52
CA characteristics					
Initial CA rhythm, n (%)	8		20 (100)		0.81
Asystole		24 (63)	12 (60)	12 (67)	
VF		10 (26)	6 (30)	4 (22)	
PEA		4 (11)	2(10)	2(11)	
Witnessed cardiac arrest, n (%)	0	12 (26)	4 (15)	8 (42)	0.04
CPR duration in prehospital^b^ (min), median (IQR)	12	54 (34-100)	84 (47-100)	40 (30-103)	0.44
Defibrillation attempts^c^, n (%)	0	22 (48)	16 (59)	6 (32)	< 0.01
Varia					
Temperature (°C), mean (SD)	10	23.7 (4.1)	23.0 (4.3)	24.8 (4.1)	0.20
Time to hospital (min), median (IQR)	11	69 (51-120)	73 (51-120)	66.5 (49.5-129)	0.78
Prehospital intubation, n (%)	2	44 (100)	26 (100)	18 (100)	-

The “missing” column corresponds to the number of missing values per variable among the 46 patients

*Abbreviations*: *CA* cardiac arrest, *CPR* cardiopulmonary resuscitation, *IQR* interquartile range, *PEA* pulseless electrical activity, *SD* standard deviation, *VF* ventricular fibrillation

^a^Variables included in the HOPE score calculation

^b^Duration from initiation of CPR in the prehospital field to hospital admission

^c^Number of shocks administered: 1 (*n*=2), 2 (*n*=3), 3 (*n*=5), 4 (*n*=3), 5 (*n*=2), 6 (*n*=1), 7 (*n*=3), 12 (*n*=1)

**Table 2:** Patient characteristics at hospital admission based on patient records (*n*=46)

	Missing	Overall (*n*=46)	University Hospital (*n*=27)	Regional Hospital (*n*=19)	*p*-value
Varia					
Temperature^a^ (°C), mean (SD)	0	24.0 (3.7)	23.3 (3.6)	24.9 (3.7)	0.10
CA characteristics					
CA rhythm, n (%)	3				0.60
Asystole		31 (72)	18 (72)	13 (72)	
VF		9 (21)	6 (24)	3 (17)	
PEA		3 (7)	1 (4)	2 (11)	
Blood gas analysis					
Potassium^a^ (mmol/L), median (IQR)^b^	0	5.2 (3.9-7.9)	5.1 (3.9-8.0)	5.5 (4.0-7.1)	0.72
pH, median (IQR)	2	6.89 (6.64-7.12)	6.95 (6.78-7.12)	6.66 (6.46-7.04)	0.10
PaCO_2_ (mmHg), median (IQR)	6	50.3 (32.3-75)	44.3 (28.5-69.8)	54 (39.8-75.8)	0.34
PaO_2_ (mmHg), median (IQR)	7	60.8 (24-124.5)	37.5 (22.5-78.8)	82.5 (38.3-198.8)	0.03
HCO_3_ (mmol/L), median (IQR)	5	8.5 (4.6-11.9)	8.7 (5.9-12.9)	7.4 (4-11.9)	0.58
Lactate (mmol/L), median (IQR)	3	14 (6-22)	7 (11-15)	16 (6-26)	0.17
Haemoglobin (g/L), median (IQR)	3	139 (115-159)	139 (114-158)	142 (115-159)	0.95
ECLS					
Type of ECLS					0.81
VA-ECMO		30 (65)	18 (67)	12 (63)	
CPB		16 (35)	9 (33)	7 (37)	
CPR duration^a^ (min), median (IQR)^c^	1	98 (77-140)	101 (86-160)	95 (70-139)	0.65

The “missing” column corresponds to the number of missing values per variable among the 46 patients

*Abbreviations*: *CA* cardiac arrest, *CPB* cardiopulmonary bypass, *CPR* cardiopulmonary resuscitation, *IQR* interquartile range, *ECLS* extracorporeal life support, *HCO*_*3*_ bicarbonate, *PaCO*_*2*_ partial pressure of carbon dioxide, *PaO*_*2*_ partial pressure of oxygen, *PEA* pulseless electrical activity, *SD* standard deviation, *VA-ECMO* venoarterial extracorporeal membrane oxygenation, *VF* ventricular fibrillation

^a^Variables included in the HOPE score calculation

^b^Measurement of potassium was obtained by arterial sampling (except for 2 patients with venous sampling)

^c^CPR duration is defined as the time from initiation of CPR in the prehospital setting until initiation of ECLS

**Fig. 2:**
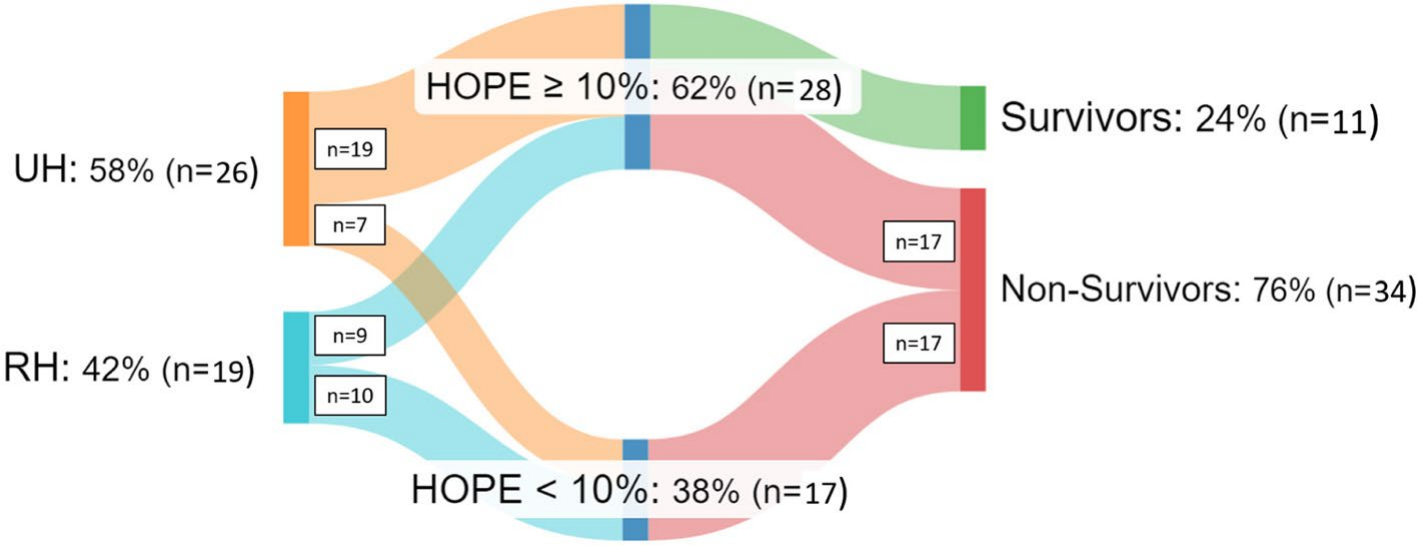
Eligibility for ECLSR by using the HOPE score and outcome comparison between medical centers (*n*=45). The HOPE score was not calculated for 1 patient at UH due to missing data and was further excluded for analysis. Abbreviations: UH, University Hospital; ECLSR, extracorporeal life support rewarming; HOPE, hypothermia outcome prediction after ECLS; RH, Regional Hospital

The potassium value at hospital admission would have contraindicated ECLS rewarming for 4 patients (8%), all of whom died (*n*=3, RH, and *n*=1, UH; *p*=0.21). A HOPE score <10% would have contraindicated ECLS rewarming, and therefore prevented futile rewarming for 17 of the 46 patients with successful ECLS implementation (37%). All survivors (*n*=11, 100%) had a HOPE score ≥ 10%, in favour of ECLS rewarming (Fig. 2). Considering only those patients in whom ECLS implementation was successful, survival was 24% (11/46); 33% at the UH (9/27) and 11% at the RH (2/19); *p*=0.07 (Table 3).

**Table 3:** Hospital outcomes of hypothermic cardiac arrest successfully rewarmed with ECLS (*n*=46)

	Missing	Overall (*n*=46)	University Hospital (*n*=27)	Regional Hospital (*n*=19)	*p*-value
VA-ECMO duration^a^ (hours), median (IQR)	2	91 (42-151) (*n*=15)	41 (27-72) (*n*=6)	125 (91-180) (*n*=9)	< 0.01
CBP duration^b^ (hours), median (IQR)	2	120 (100-150)	106 (91-130)	132 (100-189)	0.61
ICU duration^c^ (hours), n (%)	0				0.01
<=24, n (%)		10 (40)	2 (15)	8 (67)	
24-72 hours		3 (12)	1 (8)	2 (17)	
>72 hours		12 (48)	10 (77)	2 (17)	
Survival, n (%)	0	11 (24)	9 (33)	2 (11)	0.07
CPC of the survivors	1				-
1-2^d^, n (%)		10 (100)	8 (100)	2 (100)	

The “missing” column corresponds to the number of missing values per variable among the 46 patients

*Abbreviations*: *CBP* cardiopulmonary bypass, *CPC* cerebral performance category, *CPR* cardiopulmonary resuscitation, *ICU* intensive care unit, *IQR* interquartile range, *ROSC* return of spontaneous circulation, *VA-ECMO* venoarterial extracorporeal membrane oxygenation

^a^For the 17 patients who received VA-ECMO with ROSC

^b^For the 12 patients who received CBP with ROSC

^c^For the 25 patients with ROSC and ICU stay

^d^None of the survivors had a CPC score greater than 2

After exclusion of patients with unsuccessful ECLS (*n*=4) and one for whom the HOPE calculation was not feasible (*n*=1), the survival rate of the 45 remaining patients was 35% (9/26 at the UH and 11% (2/19) at the RH. The raw increase in survival rate of 35%−10.5%=24% at the UH compared to the RH was not statistically significant (*p*=0.06). Based on HOPE score survival probabilities, the expected proportion of survivors was 41% at the UH and 26% at the RH, meaning that both hospitals underperformed compared to their expected outcomes. The deficit of survivors was 41%−35%=6% (*p*=0.37) at the UH and 26%−11%=15% (*p*=0.03) at the RH. The difference between these two deficits of 15%−6%=9%, corresponding to the adjusted increase in proportion of survivors at the UH compared to the RH, was not statistically significant (*p*=0.38).

Forensic investigations were conducted for 18 patients (UH, *n*=10; RH, *n*=8). All patients received an external examination. A total of 6 received a complete forensic autopsy and 4 received a post-mortem imaging (PMCT). A summary of the findings is presented in Additional file 2.

## Discussion

We analysed a population of adult hypothermic CA patients who received ECLS rewarming, comparing outcomes between the UH and the RH. To our knowledge, this is the first study to assess the quality of care of hypothermic CA patients who received ECLS rewarming using predicted survival probabilities among a whole population.

The decision to utilize ECLS rewarming for hypothermic CA patients is critical and is often made with limited understanding of the clinical situation. Reliable prediction of survival in individual hypothermic CA patients is critical, especially to reduce the risk of underestimating the chance of survival, reducing the risk of undertreating patients with the potential to recover with good neurologic outcomes. Most of the decisions to implement ECLS rewarming in the present study were compliant with the guidelines based on serum potassium, as only 8% received ECLS despite having a potassium value higher than the maximum suggested threshold [12, 16]. The historical approach to the decision of whether to rewarm hypothermic CA patients using serum potassium alone was based mostly on observational data from historical case series and case reports [1, 17–19]. This approach, based on a single dichotomous biological criterion, has recently changed. The use of a potassium level alone is less accurate than the use of a multivariable algorithm, and is prone to preanalytical bias, as well as intraindividual variability depending on the sampling site [5, 20]. Since 2021, the ERC has recommended that in-hospital prediction of survival from hypothermic CA with extracorporeal rewarming should be based on multivariable scores. The only externally validated score is the HOPE score [3].

The HOPE score has been demonstrated to discriminate more accurately between survivors and non-survivors than a potassium level alone in the triage of hypothermic CA patients [5, 6]. Serum potassium favours over-triage of these patients compared to the HOPE score, meaning that using a serum potassium level alone leads to more futile attempts at resuscitation with unnecessary use of hospital resources, such as ECLS rewarming. This was also illustrated in a recent study that analysed hypothermic CA patients who were not rewarmed by ECLS, despite being admitted to the two same hospitals equipped for it. In this study, 54% of patients who had a potassium value not contraindicating ECLS rewarming would have not been rewarmed by using a HOPE score <10% [21]. The use of the HOPE score in our retrospective study would have prevented 37% of futile rewarming attempts without any additional loss of life, since 100% of the survivors who were rewarmed with ECLS had a HOPE score >10%. This demonstrated the superiority of the HOPE score over potassium alone in predicting the survival of hypothermic CA patients.

Our study found unfavourable differences between expected and observed survival rates. The expected survival probabilities based on the HOPE score were higher than the actual survival rates of our study population for both the UH and RH, indicating underperformance of both hospitals with respect to the expected survival. Unlike at the UH, this deficit observed at the RH was statistically significant. Gross survival rate was 24% lower at the RH compared to the UH (p=0.06), but this difference was only 9% and far from statistical significance after adjustment for the expected survival rates. However, given the differing patient populations presenting to the two hospitals, this comparison may be difficult to interpret. A significant difference between expected and observed survival would have raised questions about potential key factors impacting the outcomes at each hospital. Various hypotheses could explain a discrepancy, including the relatively low annual incidence of hypothermic CAs presenting to both hospitals and the challenging and advanced medical skills and procedures required to achieve good outcomes, especially the use of VA-ECMO [15]. Regarding the type of ECLS, the majority (65%) of our rewarmed patients received ECMO, with a higher proportion of CPB at the RH than the UH. However, the HOPE derivation study found no association between the type of ECLS and mortality rate [5]. For hypothermic CA patients, ECMO is still considered the preferred method due to its lower anticoagulation requirements than CPB and its potential for prolonged circulatory support [15].

The Extracorporeal Life Support Organization recommends that at least 20 ECMO procedures be performed annually per hospital to maintain a high level of competence and optimise outcomes [22–24]. This recommendation is based on data showing that higher-volume centres tend to achieve lower 30-day in hospital mortality, 1-year mortality, and lower rates of complications, independently of the indication for ECMO [23, 25–27]. The volume of ECMO procedures per hospital may partially explain the observed difference in survival rates between the UH and the RH.

Predictive models are important, not only for clinical decision-making and resource allocation, but also for assessing quality of care and allow for benchmarking [28]. Numerous mortality prediction tools are used daily in emergency and intensive care medicine, not only on the individual level, but also to assess groups of patients [29, 30]. Examples include the Trauma Score and Injury Severity Score (TRISS), used to estimate the expected mortality in trauma based on anatomical and physiological data, the mechanism of injury, and the patient’s age, and the Survival after Veno-arterial ECMO score (SAVE), used to predict survival after VA-ECMO for refractory cardiogenic shock [31–33]. The estimation of a standardized mortality ratio (ratio between the observed and predicted mortality) in a population allows for benchmarking and assessing the quality of care, allowing for inter-hospital comparisons [29].

The use of the HOPE score with an adjusted analysis in our study enabled better and more equitable hospital benchmarking and evaluation of hospital results, compared with the sole analysis of gross survival rates, particularly with the varied case-mix distribution of hypothermic CA patients between the two hospitals. The proportion of asphyxia-related cases was significantly higher at the RH (53%) due to its proximity with Swiss Alps and significantly higher number of avalanche victims. Avalanche victims commonly suffer from asphyxia, hypoxia, and traumatic injuries in addition to hypothermia. These conditions are associated with worse outcomes in CA. This could partly explain the difference between the expected and the actual survival reported for our patients [34–36].

### Limitations

The main limitation is the retrospective design of the study requiring manual data extraction. We had to rely on the quality of documentation from prehospital and in-hospital records. We found an overall ECMO implementation failure rate of 8% and excluded patients in whom ECMO failed from the overall estimation of survival using the HOPE score. This was intentional, as the HOPE score was derived and validated in a population of hypothermic CA patients who were rewarmed with ECLS, and therefore did not include patients for whom ECLS failed [5, 6]. This may have skewed the survival statistics. Another limitation is the size of the studied population over a long study period of more than 22 years. Finally, the ability to generalize our results may be limited by the specific features and characteristics of the two hospitals we studied.

## Conclusion

The survival of patients with hypothermic CA depends on the success of ECLS implementation, as well as patient characteristics on admission. The HOPE score can be used to estimate an individual’s expected survival probability, with a recommended threshold for ECLS rewarming when the HOPE is ≥ 10%. The HOPE score outperformed serum potassium alone in terms of appropriate triage and selection of patients for ECLS rewarming. The use of the HOPE score in a population of patients rewarmed with ECLS may help to estimate expected survival probabilities for cohorts of patients, enabling internal quality improvement and comparison of outcomes among ECLS centres.

## Supplementary Information

Supplementary Material 1.

Supplementary Material 2.

## Data Availability

No datasets were generated or analysed during the current study.

## Abbreviations

CA: Cardiac arrest
CPB: Cardiopulmonary bypass
CPC: Cerebral performance categories
CPR: Cardiopulmonary resuscitation
ECLS: Extracorporeal life support
ECMO: Extracorporeal membrane oxygenation
ERC: European Resuscitation Council
HOPE: Hypothermia outcome prediction after ECLS
ICU: Intensive care unit
PMCT: Post-mortem whole body computed tomography
ROSC: Return of spontaneous circulation
RH: Regional hospital
UH: University hospital
VA-ECMO: Venoarterial ECMO
