# Supplementary material for: Use of the HOPE score to assess survival outcome of hypothermic cardiac arrest selected by ECLS rewarming
Source: Scand J Trauma Resusc Emerg Med. 2025 Jul 28;33:132. doi: 10.1186/s13049-025-01445-9 (PMC12305985; doi:10.1186/s13049-025-01445-9)
Supplement: Supplementary file 1 — Supplementary Material 1. [file 13049_2025_1445_MOESM1_ESM.docx]

**Additional file 1. Supplementary prehospital characteristics of patients who received ECLS rewarming (n=46).** Abbreviation: IQR, interquartile range.

|  | **Missing** | **Overall**  **(n=46)** | **University Hospital**  **(n=27)** | **Regional Hospital**  **(n=19)** | **p-value** |
| --- | --- | --- | --- | --- | --- |
| **Mechanism** n (%) | 0 |  |  |  | 0.02 |
| Exposure |  | 24 (52) | 17 (63) | 7 (37) |  |
| Avalanche |  | 13 (28) | 3 (11) | 10 (53) |  |
| Submersion |  | 7 (15) | 6 (22) | 1 (5) |  |
| Immersion |  | 2 (4) | 1 (4) | 1 (5) |  |
| **CA characteristics** |  |  |  |  |  |
| Epinephrine administration, n (%) | 15 | 29 (66) | 17 (68) | 12 (63) | 0.74 |
| Epinephrine dose prehospital (mg), median (IQR) | 20 | 5 (2-8) | 7 (3-10) | 3.5 (1.5-6) | 0.07 |
| **Varia** |  |  |  |  |  |
| Mean of transportation, n (%) | 0 |  |  |  | 0.70 |
| Helicopter |  | 35 (76) | 20 (74) | 15 (79) |  |
| Ambulance |  | 11 (24) | 7 (26) | 4 (21) |  |
