# Supplementary material for: Use of the HOPE score to assess survival outcome of hypothermic cardiac arrest selected by ECLS rewarming
Source: Scand J Trauma Resusc Emerg Med. 2025 Jul 28;33:132. doi: 10.1186/s13049-025-01445-9 (PMC12305985; doi:10.1186/s13049-025-01445-9)
Supplement: Supplementary file 2 — Supplementary Material 2. [file 13049_2025_1445_MOESM2_ESM.docx]

**Additional file 2.** Results and conclusions of the forensic investigations for patients who died after ECLS rewarming (n=18). Abbreviations: ECLS, extracorporeal life support.

| **Detailed findings from forensic investigations including autopsy, external and radiological examinations** | **n** |
| --- | --- |
| **Type of forensic investigations** | |
| External examination | 18 |
| Autopsy | 6 |
| Post-mortem whole body computed tomography (PMCT) | 4 |
| **Cause of death determined** | **15** |
| Mechanical asphyxia | 6 |
| Polytrauma | 4 |
| Drowning | 3 |
| Accidental hypothermia by cold exposure | 2 |
| **Undetermined** | **3** |
